# Conservation of structure and activity in *Plasmodium *purine nucleoside phosphorylases

**DOI:** 10.1186/1472-6807-9-42

**Published:** 2009-07-03

**Authors:** Apirat Chaikuad, R Leo Brady

**Affiliations:** 1 Department of Biochemistry, University of Bristol, Bristol, BS8 1TD, UK; 2 Current address: Oxford Structural Genomics Consortium, Oxford, UK

## Abstract

**Background:**

Purine nucleoside phosphorylase (PNP) is central to purine salvage mechanisms in *Plasmodium *parasites, the causative agents of malaria. Most human malaria results from infection either by *Plasmodium falciparum (Pf)*, the deadliest form of the parasite, or by the widespread *Plasmodium vivax (Pv)*. Whereas the PNP enzyme from *Pf *has previously been studied in detail, despite the prevalence of *Pv *little is known about many of the key metabolic enzymes from this parasite, including *Pv*PNP.

**Results:**

The crystal structure of *Pv*PNP is described and is seen to have many features in common with the previously reported structure of *Pf*PNP. In particular, the composition and conformations of the active site regions are virtually identical. The crystal structure of a complex of *Pf*PNP co-crystallised with inosine and arsenate is also described, and is found to contain a mixture of products and reactants – hypoxanthine, ribose and arsenate. The ribose C1' in this hybrid complex lies close to the expected point of symmetry along the PNP reaction coordinate, consistent with a conformation between the transition and product states. These two *Plasmodium *PNP structures confirm the similarity of structure and mechanism of these enzymes, which are also confirmed in enzyme kinetic assays using an array of substrates. These reveal an unusual form of substrate activation by 2'-deoxyinosine of *Pv*PNP, but not *Pf*PNP.

**Conclusion:**

The close similarity of the *Pf *and *Pv *PNP structures allows characteristic features to be identified that differentiate the *Apicomplexa *PNPs from the human host enzyme. This similarity also suggests there should be a high level of cross-reactivity for compounds designed to inhibit either of these molecular targets. However, despite these similarities, there are also small differences in the activities of the two *Plasmodium *enzymes.

## Background

**Figure 1:**
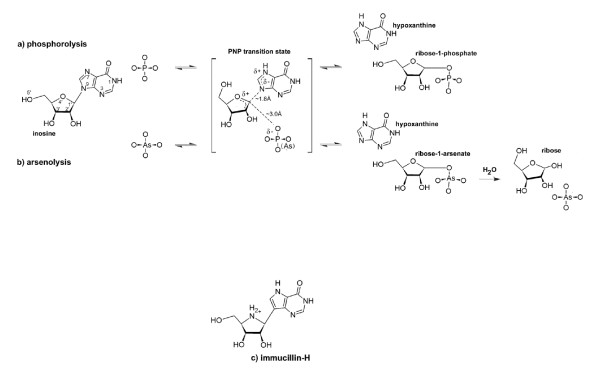
**Catalysis by PNP**. Schematic diagrams showing the chemical reactions catalysed by PNP: (a) phosphorolysis, and (b) arsenolysis. These diagrams are based on arsenolytic/hydrolysis transition-state structures of PNPs [17,3,4]. Note protonation at N7, leading to formation of a positive charge, and glycosidic-bond cleavage resulting in formation of a ribo-oxocarbenium ion and negative charge in the purine ring at the transition state. This process occurs prior to nucleophilic attack as the catalytic reaction of PNP follows a SN1 type mechanism. (c) shows the transition state mimc inhibitor, Immucillin H (ImmH).

Genomic studies [1] of the Apicomplexa parasite *Plasmodium falciparum *– the causative agent of life-threatening malaria – have confirmed earlier observations that this parasite lacks metabolic pathways for *de novo *synthesis of purines, and hence that purine salvage is essential for their survival. Inhibitors that block recycling of purines should therefore form a viable basis for novel malarial therapeutics. Purine nucleoside phosphorylase (PNP) in *Plasmodium *forms a key enzyme in the recycling of predominantly host-derived purines, catalysing the phosphorolysis of inosine to produce the major purine precursor for the salvage pathway, hypoxanthine, and ribose-1-phosphate (Figure 1a). PNP also catalyses phosphorolysis of methylthioinosine, and hence is believed to play an important role in the recycling of this purine from the polyamine biosynthesis pathway [2]. Although unlikely to be of direct biological relevance, arsenate can replace phosphate in this reaction generating ribose-1-arsenate, which is then rapidly and irreversibly hydrolysed to ribose and arsenate (Figure 1b). This alternative reaction is of interest to dissect the mechanistic details of PNP and has contributed to inhibitor development. Kinetic isotope (KIE) studies have been used to study the PNP mechanism in detail [3,4] and indicate that catalysis proceeds via a classic SN1 nucleophilic substitution reaction in which, at the transition state, the ribitol ring forms an oxocarbenium ion (Figure 1a). The derived conformation of this ring has been exploited in the design and synthesis of tight-binding and specific PNP inhibitors such as Immucillin-H (ImmH) (Figure 1c). ImmH is believed to mimic the transition-state formed during this reaction, and binds to the human form of the enzyme (hPNP) with higher affinity (K_d _= 56 pM) than do either the reaction substrate inosine (K_M _= 40 μM) or product hypoxanthine (K_M _= 10 μM). Binding of ImmH to PNP has been studied in much detail and crystal structures of its complexes with a range of PNP enzymes including bovine PNP (bPNP, together with PO_4_, [5]) and *Plasmodium falciparum *PNP (*Pf*PNP, together with SO_4_, [6]) have been reported to resolutions of 1.5 Å and 2.2 Å respectively. *In vivo *genetic knock-out studies have recently confirmed *Pf*PNP as the molecular target for the anti-parasiticidal activity of this family of compounds [7].

Crystal structures and sequence homology studies have distinguished two broad families of PNP enzymes: those that form trimers (such as the mammalian PNPs, Type 2) and those that are hexameric (such as *E.coli *PNP (EcPNP), Type 1). These groupings correlate with functional differences in that the hexameric enzymes have wider substrate specificity, in particular the ability to use 6-aminopurine nucleosides as substrates. Using phylogenetic studies [8] showed that the *Plasmodium *PNPs are outliers with equal genetic distance between PNPs and uridine phosphorylases. Crystallographic studies of *Pf*PNP [6,9] have shown it forms a hexameric assembly suggesting its closer alignment with the Type 1 PNP group, although functionally it has been noted that, unlike other hexameric PNPs, adenosine is not a substrate for *Pf*PNP. These distinguishing features suggest selectivity for *Pf*PNP, in preference to its mammalian homologues, should be achievable in the development of novel anti-malarials. However, the generality of these distinguishing PNP features across *Plasmodium *parasite species remains unclear. A recent crystal structure of PNP from the closely related simian parasite *Plasmodium knowlesi *(*Pk*PNP) has also been determined (entry 2b94 in the Protein Data Bank (PDB)) and, although forming a similar hexameric arrangement, the arrangement of the subunits differs from *Pf*PNP. This largely arises as three loop regions at the subunit interface have been traced to different conformations. In addition, the arrangement of residues in the substrate binding pocket – which does not contain substrate and adjoins one of the loops and the subunit interface – differs significantly in the *Pk*PNP structure relative to *Pf*PNP. It is therefore unclear whether *Pf*PNP represents an archetypal or unique member of the *Plasmodium *PNP enzyme family.

In this study we have therefore extended studies of *Plasmodium *PNPs, firstly through a structural analysis of PNP from the second most prevalent human-specific malaria parasite, *Plasmodium vivax *(*Pv*PNP). Malaria due to *Plasmodium vivax *infection accounts for up to 40% of the annual incidence of the disease [10] and, although generally less severe, causes considerable morbidity. A genomic sequence for *Plasmodium vivax *has recently become available [11]. *Pv*PNP and *Pf*PNP share 81% amino acid sequence identity. Secondly, although the enzyme mechanism involving generation of a ribitol oxocarbenium ion is believed to be general for all PNPs, the identity, contribution and movements of amino acids within the active site during catalysis differs between various forms of PNP. To further mechanistic understanding of the unusual group of *Plasmodium *PNPs, we have also determined the crystal structure of the arsenolytic complex of *Pf*PNP with inosine which is found to contain a mixture of product and reactant components from the reaction. Parallel kinetic and binding studies are further used to pinpoint *Plasmodium*-specific features of PNP.

## Results

### Crystal structure of *Pv*PNP

#### Overall structure

**Figure 2:**
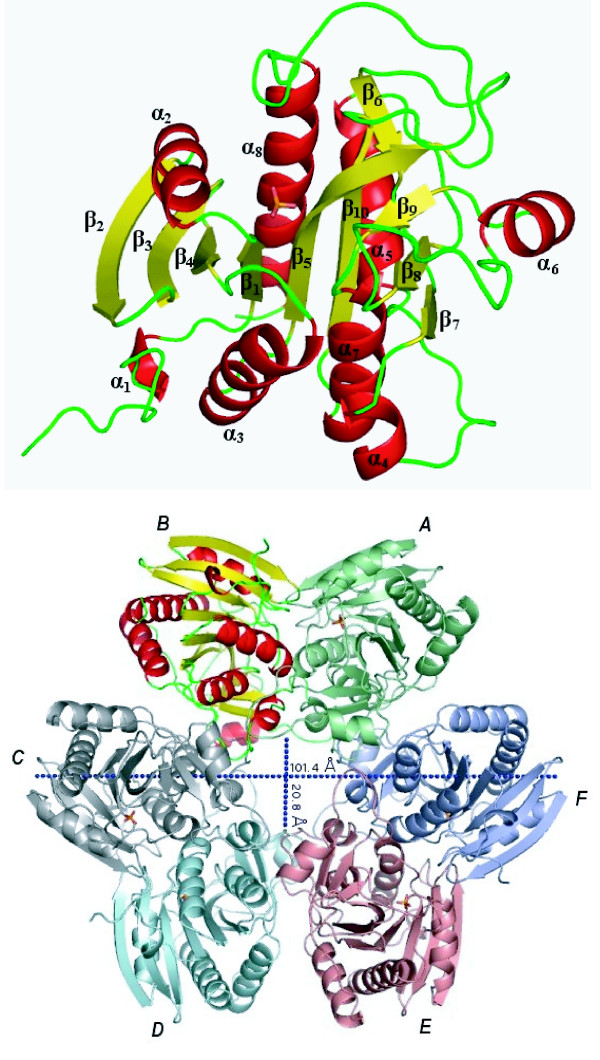
**Overall structure of *Pv*PNP**. The ribbon diagram shows a monomer of *Pv*PNP with the secondary structure elements labelled. The bottom panel shows the assembled hexamer with each subunit in a different colour, viewed perpendicular to the three-fold axis.

The *Pv*PNP crystal structure was refined against 1.85 Å resolution data (summarised in Table 1) and the model contains all of the protein residues with the exception of the disordered active site loop (residues 212–224). The R32 crystals displayed considerable anisotropy in the distribution of their diffraction intensities leading to the rejection of many higher resolution reflections during processing. Nonetheless, about 70% of the processed reflections have intensities with I/(sigma I) greater than 3 in the highest resolution shell, hence the structure has been refined against all available data to 1.85 Å. The crystallographic asymmetric unit contains a monomer of *Pv*PNP which adopts the familiar single-domain fold topology described previously for hexameric PNPs from other species (e.g. [6,9,12,13]). Each *Pv*PNP monomer is comprised of a 10-stranded *β*-sheet core, which forms the base of the catalytic site, and eight *α*-helices, which are involved in subunit contacts (labelled in Figure 2). The total number and position of secondary structure elements in *Pv*PNP are comparable to those in *Pf*PNP, although small variations in the number of *β*-strands and *α*-helices are evident due to different assignments when comparing *Pf*PNP structures ([9,6] and the *Pf*PNP solved in this study). An extra *α*-helix in the ordered active site loop of *Pf*PNP is not observed in the *Pv*PNP structure, but may be formed when the *Pv*PNP active site loop is structured.

**Table 1:** Summary of crystallographic data.

	*PvPNP*	*Pf*PNP hypoxanthine-ribose-AsO_4 _complex
PDB code	3EMV	3ENZ

Space group	R32	I4_1_22

Unit cell	a = b = 94.8 Å,	a = b = 177.8 Å, c = 253.9 Å
	c = 121.3 Å	α = β = γ = 90.0°
	α = β = 90.0°,	
	γ = 120.0°	

Resolution range (Å)	33.98 – 1.85 (1.92 – 1.85)	30.94 – 2.03 (2.10 – 2.03)

Unique reflections	14,619 (875)	129,248 (12,783)

Completeness (%)	80.5 (49.0)	99.4 (99.8)

Redundancy	6.0 (4.4)	10.7 (8.0)

I/σI	17.6 (4.3)	18.5 (2.7)

R_*merge*_	0.095 (0.234)	0.106 (0.556)

Solvent content	33.5%	58.9%

***Refined model***		

No. Protein chains	1	6

No. Protein atoms	1921	11268

No. Other atoms (ligand, organic solvent and water)	80	779

No. TLS groups	1	30

R_fact_	0.193	0.160

R*free*	0.233	0.193

FOM	0.852	0.891

Mean B_*fac *_protein (Å^2^)	25.7	16.1

Mean B_*fac *_other atoms (Å^2^)	29.6	28.8

rms bonds (Å)	0.012	0.016

rms angles (°)	1.611	1.396

Table shows processing and refinement statistics for the diffraction data and models from both crystal forms. The numbers in parentheses refer to data from the highest resolution shell.

The nucleoside binding site of the *Pv*PNP enzyme in this structure is observed to be empty although there is electron density consistent with three bound water molecules within the substrate binding site. Additionally, an anion – presumably sulphate from the crystallisation buffer which contained 0.2 M LiSO_4 _– was identified and this anion occupies the phosphate binding pocket of the enzyme. Its position is consistent with sulphate and phosphate groups observed in other PNP structures including *Pf*PNP [6,9].

When the *R*32 symmetry operators are applied, *Pv*PNP is seen to form a hexameric assembly that matches the arrangements previously reported in crystal structures of *Pf*PNP [6,9] and *Ec*PNP [14]. Presumed to be the biologically-relevant structure, this hexamer is a disc-shaped single-layer with an overall diameter of about 100 Å and a thickness corresponding to the width of a monomer (approximately 50 Å) with an empty central channel of diameter 20 Å. The hexameric *Pv*PNP appears to be assembled from a trimer of dimers, where the dimer pairs are related by a crystallographic three-fold symmetry axis running through the central channel, corresponding to the view in Figure 2. The two monomers in each pair of dimers lie anti-parallel and are related by a crystallographic two-fold axis perpendicular to the major three-fold axis, with the two active sites lying 22 Å distant from each other.

Inter-subunit contacts in *Pv*PNP are comparable to those reported in the previous *Pf*PNP-inosine structure [9] but different from those observed in the *Pk*PNP structure [PDB:2B94]. The loop (residues 159–170) that connects *β*_8 _and *α*_6 _is particularly evident in its contribution to the subunit interactions. This loop is adjacent to the central channel and forms contacts both with the neighbouring subunit in each dimer pair – such as A and B – and also extends to a monomer of the adjacent dimer – such as A and C. In the former case, a distinctive hydrogen bond is formed between Tyr 162 of chain A and Glu 78 of chain B, which has also been described for *Pf*PNP [9] and PNP from *Thermus thermophilus *(*Tt*PNP) [15]. The length of this loop differs between hexameric PNP enzymes from different species. Relative to *Pk*PNP and *Tt*PNP, the *Pv *and *Pf *enzymes have loops extending about 11 Å further from the core, whereas in *Ec*PNP the equivalent loop extends about 6 Å. In the *Pf*PNP-inosine structure, Schnick *et al*. [9] showed that this loop also contacts the ligand and proposed that this elongated loop plays an important role for not only quaternary structure formation but also in determining accessibility to and the conformation of the active site cavity. With no substrate bound in the current structure it is not possible to confirm this is also the case for the *Pv*PNP enzyme.

#### The active site of *Pv*PNP

**Figure 3:**
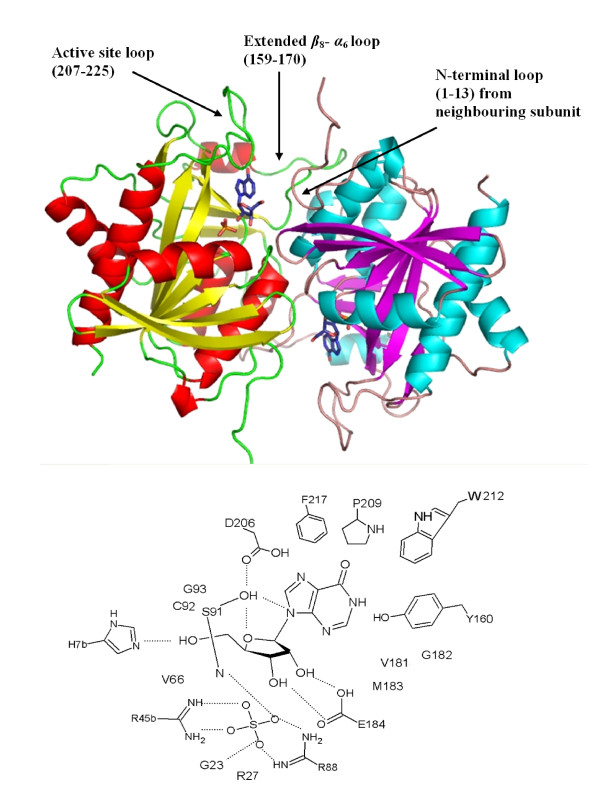
**The active site of the *Pv*PNP**. The top panel illustrates the dimer pair and their binding sites with the bound sulphate anions (from the crystal structure) and the inosine substrates (modelled into the *Pv*PNP structure based on superimposition of the *Pf*PNP-inosine structure, PDB:2BSX). The bottom schematic diagram shows possible key residues of the *Pv*PNP active site (numbered according to the system previously used for *Pf*PNP) with hydrogen bonds as dashed lines. Most residues are from the parent monomer, while those labelled 'b' are from the neighbouring subunit across the dimeric surface.

In common with *Pf*PNP, the phosphate/sulphate binding site of *Pv*PNP (Figure 3) is formed mainly by two arginine residues (Arg 89 and Arg 46' from the neighbouring monomer), a backbone interaction with Gly 24, and both backbone and side chain interactions with Ser 91. Another arginine, residue 27, also lies in this region, but points away from the bound sulphate in the *Pv*PNP structure. This is similar to the arrangement in *Pf*PNP with sulphate bound [9] but differs from *Pf*PNP with arsenate bound (see below). Overall, the PO_4_/SO_4 _binding pocket in *Pv*PNP is essentially identical to that previously described for *Pf*PNP.

Although neither base nor sugar are bound in the nucleoside binding site of the *Pv*PNP-SO_4 _structure, these sites can be readily identified by overlaying the hypoxanthine and ribose sugar molecules from the *Pf*PNP-hypoxanthine-ribose-AsO_4 _structure (see next section). This shows that the base would be primarily accommodated via π-stacking of Tyr 161 and Pro 210, and also Trp 213 when the active site loop (207–225) is ordered. Other significant contributors include Ser 92, Val 182, Cys 93 and Gly 94. Asp 207, which has been proposed to play an important role in stabilisation of the transition state complex [6,16] is also present in the *Pv*PNP purine-binding site, although in a conformation typical of an empty binding site [9]. The composition and structure of the base binding pocket in *Pv*PNP is essentially identical to that described for *Pf*PNP, whereas there are substantial differences when compared to this region in the *Pk*PNP structure. Although the identity of the key residues is unaltered in *Pk*PNP, the altered conformation of the 159–170 loop results in a substantial (4 Å) displacement of Tyr 157 which, along with Tyr158 (equivalent to Tyr 161 and Tyr 162 in *Pv*PNP), now occupies the space in which the base normally binds. This leads to considerable distortion of the base binding pocket.

Similarly, the sugar binding site of *Pv*PNP is virtually identical to that of *Pf*PNP. By analogy with the *Pf*PNP complex, the sugar O2' and O3' hydroxyl groups are expected to interact with the side chains of Arg 89 and Glu 185. His 8' from the neighbouring monomer is in a position to bind the ribose O5' hydroxyl group and Ser 92 is positioned to hydrogen bond to the ribosyl ring O4'. Other residues such as Tyr 161, Met 184 and Val 67 also line the cavity in both structures. By contrast, although many of the above interactions are also possible in the *Pk*PNP structure, the altered conformation reported for the 159–170 region once again leads to changes in the sugar binding pocket, with His 8' considerably displaced and its equivalent position now occupied by Lys 164. Tyr 160, as discussed above, is present but displaced by about 4 Å and there is no direct equivalent to Val 67, although its role may be partially replicated by Ala 66.

### Crystal structure of *Pf*PNP complexed with hypoxanthine, ribose and arsenate

This structure was obtained by co-crystallising *Pf*PNP with inosine and arsenate to produce a *Pf*PNP, hypoxanthine, ribose and arsenate complex (*Pf*PNP-HRA) (see below). The asymmetric unit consists of one hexamer of *Pf*PNP in which there are six very similar, but not identical, subunits. The crystal packing differs from previously reported structures for *Pf*PNP [6,9] although the arrangement of monomers is very similar. All monomers in the hexamer have essentially the same overall secondary structure (root mean square deviations (rmsd) of equivalent Cαs between monomers range from 0.16–0.40 Å) with the exception of only one area. This is in the active site loop (206–224), part of which forms a short helix in four monomers (A, B, D and E) whereas this is not present in the remaining two chains (C and F).

#### The active site

Although inosine and arsenic acid were included in the crystallisation mixture with *Pf*PNP, the resulting electron density was inconsistent with inosine being present in the active site. The PNP-catalysed arsenolysis reaction is similar to phosphorolysis, however the ribose-1-arsenate product is unstable and is rapidly hydrolysed into ribose and arsenate [17] as shown in Figure 1.

In accordance with the shapes, sizes and positions of electron density observed within the active site, the products hypoxanthine base, ribose sugar and arsenate anion (AsO_4_) – formed from the irreversible arsenolysis of inosine followed by hydrolysis of the ribose-1-arsenate – were modelled in to the observed electron density map. These proved to be a good match for the density and were well-behaved in subsequent refinement. Based on the known sequential mechanism for release of the products [18], the inclusion of the arsenate group correlates with the presence of the ribose sugar which consecutively matches the presence of hypoxanthine. These product and reactant molecules are assumed to be accommodated within the active site in a similar way to enzyme intermediates in accordance with the observed catalytic conformation of the enzyme, including a closed active site loop with the characteristic helical segment and the side-chain conformation of Arg 27 (see below).

**Figure 4:**
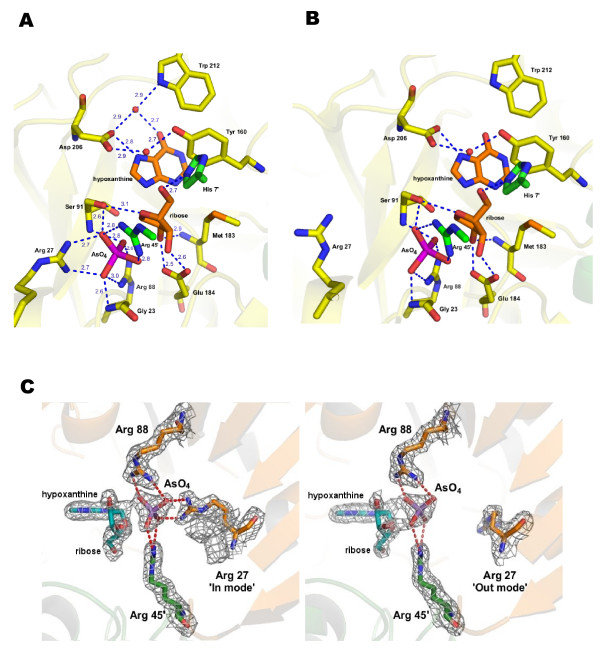
**Structure of *Pf *PNP-HRA complex**. (a) active site of typical subunit (b) active site of subunit F (c) corresponding electron density (2F_obs_-F_calc_, contoured at 1 sigma) for the 'in' and 'out' modes of Arg 27 respectively. Hydrogen bonds are shown as blue dashed lines with distances in Ångstroms, and bound water molecules are shown as red spheres.

The binding pocket is clearly defined in this structure (Figure 4). In essence, the composition of the pocket is identical to that previously described in the *Pf*PNP structures [6,9]. However, there are important differences in the conformations of several key catalytic residues.

#### Arsenate binding site

In all subunits, the arsenate moiety is stabilised mainly by two arginine side chains (Arg 88 and Arg 45' of the neighbouring subunit) and further hydrogen bonds are formed with donors from the hydroxyl group of Ser 91 and the backbone amino groups of Gly 23 and Ser 91. These interactions are similar to those observed for the bound sulphate in the *Pf*PNP-SO_4 _[9] and the *Pf*PNP-ImmH [6] structures. However the participation of the Arg 27 residue in the anion binding pocket, where its guanidinium side chain forms charged hydrogen bonds with the arsenate molecule, has not been observed in structures with sulphate bound [16,19]. Despite the inclusion of arsenic acid, this complex was crystallised under conditions heavily buffered to neutral pH (4 M sodium formate) and hence the ionisation state of the arsenate is expected to be the same as for sulphate (both are dianions at this pH, believed to be the active form in catalysis [20]). Similarly, as both crystal structures have been determined at neutral pH no alterations in the ionisation states, and hence overall conformation, of the three arginine residues that dominate the arsenate/phosphate binding site would be expected. In the *Pf*PNP-HRA complex, in five of the subunits the Arg 27 side chain points toward the active site ('closed' conformation, average torsion angle χ_1 _= 163° and χ_2 _= 175°) and forms hydrogen bonds with oxygen atoms of the arsenate ion. This conformation appears to increase ordering of the anion and leaves little unoccupied space in the binding pocket. By contrast, in chain F this arginine is in an 'open' conformation with its side chain pointing away from the active site (torsion angle χ_1 _= 178° and χ_2 _= 75°) and hence does not interact with the arsenate ion.

#### Nucleoside (ribose sugar and hypoxanthine) binding site

All six binding pockets in the hexamer bind both ribose and hypoxanthine in a similar conformation with equivalent hydrogen-bond interactions formed to residues in each active site (Figure 4). The ordered active site loop brings Pro 209, Trp 212 and Phe 217 into the hydrophobic pocket, resulting in the hypoxanthine being oriented by π-stacking and van der Waals interactions. Similar to the *Pf*PNP-Imm complex, the carboxylate group of the Asp 206 side chain interacts directly with N7 of the hypoxanthine, and has been proposed to be the general acid/base for protonation of N7 of the substrate in the transition state [14,6,16]. The conformation of this flexible Asp side chain differs from that observed in the *Pf*PNP-SO_4 _and *Pf*PNP-ino structures where Asp 206 points away from the active site and forms hydrogen bonds to the hydroxyl group of Ser 91.

Also of interest is a bound water molecule close to O6 of the purine base, which is present in all subunits with Arg 27 in the 'closed' conformation. This water molecule acts as a bridge linking Trp 212 (N_ε1_) (a residue from the active site loop), Asp 206 (O_δ1_) and O6 via a hydrogen-bond network (Figure 4), and hence may play a role in catalysis or stabilisation of the transition state. In contrast, this water molecule is not observed in chain F, consistent with a state in which the active site loop is destabilised.

The ribose sugar binds in between the arsenate anion and the hypoxanthine base with its oxygen atoms (both hydroxyl oxygens and O4') fully engaged with hydrogen bonds formed with several residues (Figure 4). The interactions in this region are not significantly different from those previously described in the *Pf*PNP-ino [9] and the *Pf*PNP-ImmH [6] structures. There appear to be no conformational alterations associated with binding of the ribose sugar, consistent with the observation in the *Ec*PNP structure [16].

In summary, the hexameric structure of the *Pf*PNP complex appears to contain two states of the enzyme. In five of the subunits (chains A – E) Arg 27 is in a 'closed' conformation and the *α*_8 _helix is formed within the active site loop. In the remaining subunit (chain F) Arg 27 is in an 'open' conformation and the bound water close to O6 is absent.

### Enzyme Kinetics

Kinetic data for *Pf*PNP and *Pv*PNP are summarised and compared with existing published data [8,21-25] in Additional File 1. These data are similar to those previously reported for *Pf*PNP [8,22] with the exception that *Pv*PNP, unusually, appears to be activated by the 2'-deoxyinosine substrate as discussed below. This increase in rate was consistently observed with both different batches of *Pv*PNP and altered concentrations of the linked enzyme, xanthine oxidase, and hence appears to be an inherent characteristic of *Pv*PNP with 2'-deoxyinosine. The *Plasmodium *PNPs are seen to display slightly better catalytic efficiency for guanosine than inosine, consistent with previous reports.

## Discussion

Reconciliation of structures derived from the arsenolytic reaction of PNP with the mechanism of phosphorolysis is complicated primarily by the spontaneous breakdown of the ribose-1-arsenate product. This leads, in this study, to a complex that contains a mixture of products (hypoxanthine), a (non-enzymatic) degradation product of the real product ribose-1-arsenate (ribose) and reactants (arsenate). The relative placement of these groups within the active site differs from previous structures of PNP complexes containing products, reactants or inhibitors.

Two different forms of the active site are observed in the *Pf*PNP-HRA complex. These are distinguished by the conformation of the active site loop, and the positioning of the side chain of Arg 27. Conformational changes in the active site loop have previously been reported as a distinguishing structural feature between the ground-state (e.g. *Pf*PNP-SO4 or *Pf*PNP-ino, loop disordered) and catalytic-state (*Pf*PNP-Imm, loop forms a short helix) of *Pf*PNP [6,9]. In the *Pf*PNP-HRA complex, although all active sites are occupied, in four subunits (A,B,D,E) the active site loop is ordered – indicative of a catalytically-active form – whereas its disorder in subunits C and F may reflect the start of a transition in which the enzyme adopts a 'relaxed state' to release the products of arsenolysis. This is supported by the conformation of the Arg 27 side chain which points away from the active site in chain F, in contrast to Chain C and the other chains where this side chain participates in binding the arsenate group (Figure 4). This has not been observed previously in *Pf*PNP-Imm structures (co-crystallised with SO_4_, [6]) although conformational alteration of an equivalent arginine side chain has previously been noted to correlate with enzymatic activity in *Ec*PNP where the 'open' position of the arginine side chain has been correlated with the enzyme in a non-catalytic state [16,19]. In the EcPNP enzyme the two conformations were also proposed as an explanation for the two different phosphate affinities observed [16].

Another feature of the *Pf*PNP-HRA active site that provides some insight into the mechanistic state reflected by the complex is the binding of Asp 206 directly to the N7 of the base, an arrangement consistent with its proposed role as the general acid/base for protonation of N7 of the substrate in the transition state [14,6,9,16]. It has been suggested that this carboxylate-base interaction is exclusive to the catalytic state structure, and is unlikely to play an important role in initial substrate binding [9]. By this criterion the conformation of Asp 206 observed in the present structure implies that this structure of *Pf*PNP represents the enzyme in a catalytic state. This is further supported by the observation of a bound water molecule close to O6 of the purine base, which is present in all subunits with Arg 27 in the 'closed' conformation. This is similar to the reported structure for *Ec*PNP where the presence and absence of the equivalent water molecule correlates with the two states for the active site loop, and has lead to the suggestion that the water molecule might act as a lubricant for the folding and unfolding of the helix in the active site loop [16].

Together, these indicators are all consistent with five subunits of the *Pf*PNP-HRA complex representing an active conformation of the enzyme in an intermediate state structure. In contrast, the remaining subunit (chain F) may represent the structure of the enzyme in a non intermediate state or, speculatively, a state prior to release of the products.

### Mechanism of *Plasmodium PNPs*

**Figure 5:**
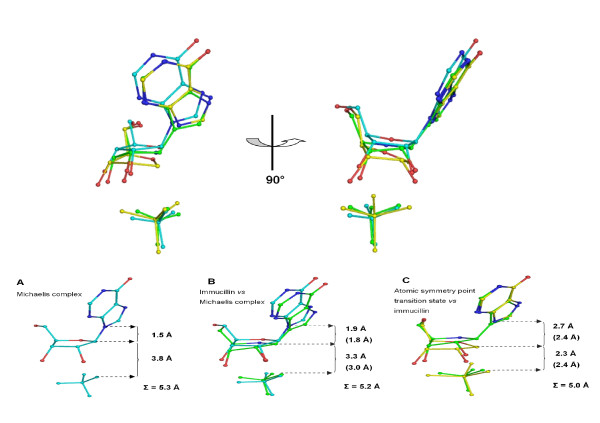
**Movement of the C1' of the ribose ring throughout catalysis by pPNP**. Top figure shows two perpendicular views of an overlay of the ribose and surrounding atoms in *Pf*PNP-inosine/*Pf*PNP-SO_4 _(Michaelis complex, green), *Pf*PNP-ImmH (transition state, purple) and *Pf*PNP-hypoxanthine-ribose-AsO_4 _complex (post-transition state, yellow) structures. Bottom panel show the distances between C1'-N9 and C1'-nucleophilic oxygen when comparing ligands in different states with the sum of the reaction coordinate distance (Σ) shown. The numbers in brackets are the values proposed by KIE calculations between the Michaelis complex and transition state [17] or those observed in bPNP crystal structures representing the transition state and post-transition state [5]. This figure is similar to those compiled previously for other PNP combinations such as in [5,26,27].

The catalytic mechanism of PNP enzymes has been dissected in detail in many previous studies. Nevertheless, the *Pf*PNP-HRA complex in this study provides an interesting addition to the many crystallographic observations that support a mechanism elucidated primarily by KIE studies. Firstly, arsenate is chemically and physically more similar to phosphate than is sulphate, which has been used extensively in many of the previous PNP crystal structures. Secondly, to form the oxocarbenium ion transition state the C1' atom of the ribose ring is required to move away from the base by about 0.3–0.4 Å [17] and then further towards the phosphate ion to form partial bonds to both the purine and the phosphate, a position known as the point of atomic symmetry in the reaction coordinate of PNP, and which is energetically similar to the complex with bound products [5]. At this point the C1' atom lies equidistant between the N9 of the base and the incoming phosphate/arsenate oxygen nucleophile. This condition is close to being satisfied in the crystal structure of the *Pf*PNP-HRA complex, with average separations of As-O1 ⋯ C1' of 2.4 Å, and C1' ⋯ N9 of 2.7 Å. By contrast, the equivalent distances in the *Pf*PNP-ImmH complex are 3.4 Å and 1.6 Å respectively, and 3.6 Å and 1.5 Å in the *Pf*PNP-ino and *Pf*PNP-SO_4 _complexes (see Figure 5, which is similar to previous figures such as in [5,26,27]). The ribose C3' atom is seen to adopt an *exo *conformation, consistent with the observation for the conformation of iminoribitol group of immucillin H in the *Pf*PNP-ImmH structure (*3*), and has an O5'-C5'-C4'-C3' dihedral angle of 174°. This differs from an earlier suggestion from KIE studies that a C3'-*endo *conformation might be adopted during catalysis (*4*). The proximity of the C1' atom to the nucleophile is consistent with a post-transition state arrangement. By contrast, *Pf*PNP-ino and *Pf*PNP-ImmH complexes are pre-transition state and (close to) transition state conformations respectively. In combination, the series of *Pf*PNP structures shown in Figure 5 provides a neat series of snapshots illustrating clearly the movement of the C1' atom of the ribose group throughout the phosphorolysis mechanism in *Pf*PNP.

Further examination of the association of the ligands with PfPNP also suggests the active site arrangement of the complex may be closer to an intermediate state rather than a straightforward product complex. Firstly, the AsO_1 _⋯ C1' distance is longer and the C1' ⋯ N9 distance is shorter than those previously described for true product complexes of mammalian PNPs (1.5 Å and 3.8 Å, respectively [28]). Further, by comparison with PNP complexes with sulphate or phosphate, the arsenate oxygen is tilted towards the ribose ring oxygen (AsO_1 _⋯ O4' = 3.2 Å) in a similar arrangement to that seen in the ImmH structure (PO_1 _⋯ N4' = 3.3 Å). This arrangement in ImmH is believed to reflect partial charge on the iminoribitol ring indicative of ribo-oxocarbenium ion character [5]. In addition, the N9 to O_1 _distance of the bound SO_4 _is 4.7 Å in the *Pf*PNP-ImmH complex, and 4.8 Å in the *Pf*PNP-HRA complex (AsO_4_). Closer distances in these complexes are generally believed to indicate more transition-like character, representative of transition state formation with significant bond order to leaving and/or attacking groups. Finally, the involvement of Arg 27 in the *Pf*PNP-HRA complex distinguishes this conformation from that previously described for the *Pf*PNP-ImmH transition-state complex.

Previous studies [16,19] have suggested that Arg 27 not only enhances the affinity of binding of the phosphate group, but also participates in catalysis by stabilising the negative charge of the anion. However, the role of Arg 27 may be more complex. Erion *et al*. [20] proposed that a basic residue in this location at this position may be involved in preparation of a catalytically active anion containing nucleophilic oxygen, by demonstrating an important role for the His 86 residue found in this location in human PNP. This histidine is believed to deprotonate the anion, hence generating the required catalytically preferred ionic state and strengthening the negative charge of the bound phosphate/arsenate anion [29]. Of the three arginines within the active sites of pPNPs, since the immobile Arg 88 and Arg 45' participate in the binding site in both the ground and intermediate states, it appears that Arg 27 may fulfil a similar role to His 86 in the human enzyme, helping to stabilise a negative charge on the phosphate anion and hence leading to activation of the nucleophile in the intermediate state. In the *Pf*PNP-HRA complex, Arg 27 forms bifurcated hydrogen bonds (average distance 2.7 Å) directly with two of the oxygens of the bound arsenate, consistent with this proposed role. This interaction is absent in the *Pf*PNP-ImmH structure, in which the arginine side chain is turned away from the bound sulphate, as also seen in one of the subunits (F) in the *Pf*PNP-HRA complex.

In discussion of the role of a similar arginine (Arg 24) in *Ec*PNP, [16] proposed that its structural rearrangement is induced by a tight-binding enzyme conformation, in which a neighbouring continuous helix is broken into two parts, one of which moves in the direction of Arg 24. This helix brings Arg 217 close to Arg 24, permitting the formation of a hydrogen bond between Arg 24 N_ε_·and the·Arg 217 main chain oxygen. This structural rearrangement is not the case for *Pf*PNP; nonetheless, stabilisation of Arg 27 is still observed but must result from a different mechanism. We note that the N_ε _atom of Arg 27 is instead surrounded by several water molecules forming bridging interactions between this N_ε _atom and the O atom of Asn 219 and O_δ2_atom of Asp 24. As phosphate and arsenate ions are similar in size and – under the crystal conditions – charged, binding of both molecules to the protein is likely to follow the same scheme.

**Figure 6:**
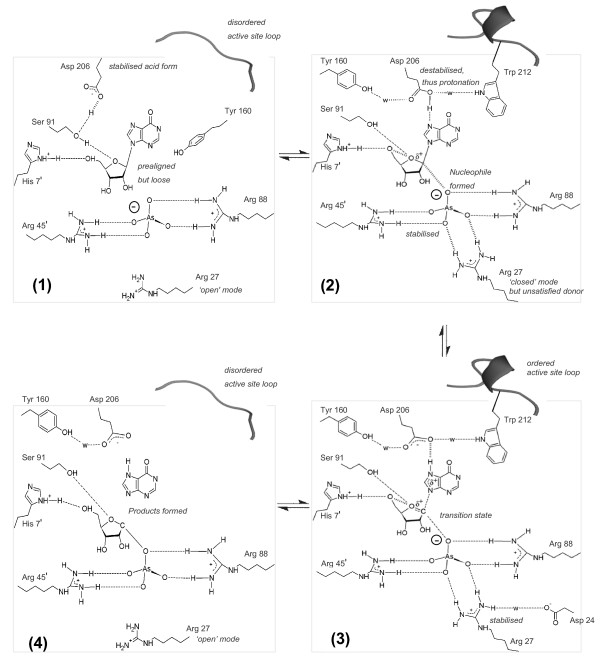
**Catalytic mechanism of *Plasmodium *PNP**. Schematic diagram showing the proposed generic *Plasmodium *PNP reaction mechanism, which is based on that initially proposed for *Ec*PNP [19]. Note that Asp 206 must be in its acidic form prior to protonation. The dotted lines indicate electrostatic interactions, dashed lines are hydrogen bonds and 'w' indicates water molecules. Panel (1) is the binding state, (2) is the pre-catalytic state, (3) is the intermediate state, and (4) is the pre-leaving state. See text for details of each step.

**Figure 7:**
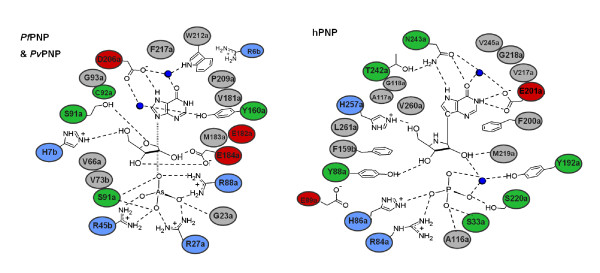
**Substrate placement and binding pocket in the *Plasmodium *and human PNP enzymes**. The schematic diagrams represent the human and parasite forms of the enzyme at the catalytic state. The diagram for *Plasmodium *parasite enzymes (left) is based on the arsenolytic-intermediate-state *Pf*PNP and the sulphate-bound *Pv*PNP structures, with the residues numbered according to the *P. falciparum *enzyme (+1 for *Pv*PNP), while that for human PNP (right) is based on the transition-state-analogue complexes (PDB ID: 1RR6, [6]) and refined atomic coordinates [20]. Amino acids lining the active sites are represented by spheres, coloured grey for non-polar, green for uncharged polar, blue for positively charged and red for negatively charged amino acids with bound water molecules shown as dark blue spheres. Note residues labelled 'a' are from the parent subunit, while those labelled 'b' from the neighbouring subunit in a dimer pair.

As the composition of the active sites is identical between the *Pv*PNP and *Pf*PNP structures, it appears reasonable to conclude that both enzymes use the same residues and have the same catalytic mechanism. Fusing a multitude of previous mechanistic studies of other PNPs with the various *Plasmodium *PNP crystal structures enables a generic mechanism for the *Plasmodium *enzymes to be summarised (Figure 6). This differs from the mammalian (trimeric) PNP mechanism primarily in the identity and contributions of several of the key catalytic amino acids. These include the anion binding site which is formed from three arginine residues in pPNPs, whereas from one arginine and one histidine in hPNP; the proton-donating residue is Asp 206 in pPNP and Asn 243 in hPNP; residues in the ribose binding pocket also differ particularly a charged Glu 184 in pPNP relative to the hydrophobic Tyr 88 in hPNP, and in pPNP there are cavities adjacent to the O5' of the ribose and N1 and C2 of the base, which are filled by hydrophobic residues for the former and Glu 201 for the latter in hPNP (Figure 7). These accumulated differences suggest that selectivity for the *Plasmodium *forms should be achievable in the design of PNP inhibitors. This has already been shown with the ImmH series of inhibitors where the derivative MT-ImmH has been reported to bind to *Pf*PNP over 100 fold tighter than to human PNP [6].

### Substrate activation in *Pv*PNP

**Figure 8:**
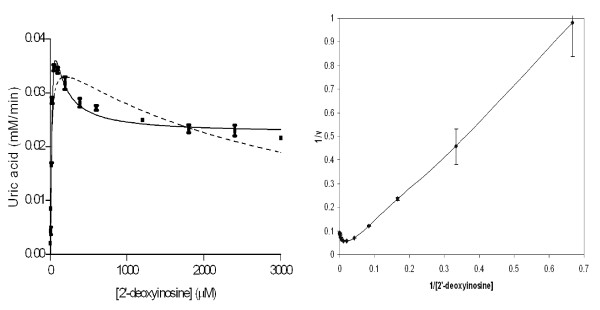
**Fitting of initial velocities to different kinetic treatment methods for catalysis of 2'-deoxyinosine by *Pv*PNP**. The left (A) shows that application of the substrate activation equation (equation 2 in the text), *v *= ((*V*_max1_·[S]) + (*V*_max2_·[S]^2^/*K*_M2_))/(*K*_M1 _+ [S] + ([S]^2^/*K*_M2_)), (**-**) matches the experimental data better than does the theoretical curve from the substrate inhibition equation (---). The reciprocal plot, 1/*v versus *1/[S], with ordinates divided by a factor of 500 (right, B) illustrate the upward curvature at high substrate concentration range, which is similar to an example of a substrate activation case given by [30].

Phosphorolysis of most nucleoside substrates by *Pf*PNP and *Pv*PNP in this study followed the typical mechanism described by the Michaelis-Menten equation – i.e. double reciprocal plots of 1/*v versus *1/[S] produce linear relationships (data not shown). However, this was not the case for the reaction of *Pv*PNP with 2'-deoxyinosine. Although 2'-deoxyinosine is unlikely to be a biologically-relevant substrate for *Plasmodium *PNP, it is similar to some of the developed PNP inhibitors such as 2'-deoxy immucillin-H and G. Substrate inhibition was first considered as an explanation for the non-linearity of the reciprocal plot for this substrate, but the data fitted poorly to the Michaelis-Menten equation corrected for substrate inhibition (*v*_0 _= *V*_max _* [S]/(*K*_M_+ [S]*(1+ [S]/*K*_i_)) (Figure 8). Plots of *v*_0 _versus log [S] (data not shown) also confirmed that curvature was not symmetrically bell-shaped, which is typical for substrate inhibition cases [30].

There have been previous reports of substrate activation of the trimeric hPNP with specific nucleoside substrates [31,32]. The data for *Pv*PNP with 2'-deoxyinosine were therefore fitted to a derived substrate activation equation following the rapid equilibrium method described by [30] and showed an improved fit (Figure 8A) leading to calculation of the kinetic parameters shown in Additional File 1. It was also noted that a reciprocal plot of 1/*v versus *1/[S] is linear over the low substrate concentration range but turns upwardly concave over a high substrate concentration range (Figure 8B). Frieden *et al *[30] noted that for substrate activation cases this curvature is normally downward, although *Pv*PNP with the 2'-deoxyinosine substrate appears to be an interesting form which has also been found in threonine dehydrase [33] and can nevertheless be classified as a substrate activation phenomenon. This distinguishes substrate activation in *Pv*PNP with 2'-deoxyinosine from other examples of substrate activation reported for other PNP enzymes [31,32] which conform to the classical substrate activation model. Application of the Hill equation to the first portion of the *Pv*PNP-2'-deoxyinosine data confirmed the existence of positive co-operativity (Hill coefficient of 1.9, data not shown), consistent with the notion of substrate activation of *Pv*PNP by 2'-deoxyinosine, a property not shared by *Pf*PNP despite the high overall structural similarity. However, the mechanism of substrate activation in PNP enzymes, particularly the unusual form observed here for *Pv*PNP, is not evident from the crystal structure. For the other substrates, the 2' hydroxyl group forms a hydrogen bond (2.5 Å) with Glu 184. The absence of this interaction when 2'-deoxyinosine is used as substrate might facilitate positioning rearrangements between the ribose and phosphate, hence explaining the increased *k*_cat_rate. Nonetheless, there is no obvious explanation in the structures as to why this capacity might vary between the *Pf*PNP and *Pv*PNP enzymes, the former not displaying substrate activation.

It is also worth noting that despite strict conservation of the active site composition and structure between the *Pf*PNP and *Pv*PNP enzymes small variations in their enzymatic activities are observed. These might arise through indirect effects from amino acid changes peripheral to the active site region, as has recently been described for mammalian PNPs [34,35]. In the assembled dimers of *Plasmodium *PNPs the amino terminus of each neighbouring subunit is located close to the adjacent subunit nucleoside binding site. This region has the greatest number of amino acid differences between the two *Plasmodium *enzymes, including a number of charge changes. These substitutions may induce electrostatic changes in the enzyme leading to alterations of the p*K*a values for the active site histidines (His 7 in *Pf*PNP or His 8 in *Pv*PNP), a mechanism that has previously been demonstrated, for example, to explain the differential activities of isoforms of human lactate dehydrogenase despite their identical active sites [36].

## Conclusion

As expected from sequence homology comparisons, the overall structure and active site of *Pv*PNP is very similar to those previously described for *Pf*PNP. However, these structures both differ from the crystal structure of *Pk*PNP in which, despite overall conservation of most active site amino acids, a considerably different active site arrangement is observed. This appears to arise from the extended 159–170 loop in *Pk*PNP which protrudes at the dimer interface, changing the subunit association in the dimer and hexamer and, in turn, the binding pocket. It is difficult to conceive, however, that the site could accommodate substrates in this form which may represent an inactive form of the enzyme. The comparability of the *Pf*PNP and *Pv*PNP structures provides confirmation that these are likely to reflect the archetypal *Plasmodium *forms of the enzyme. The close structural coincidence of their active sites, and their similar overall kinetic profiles, suggest that inhibitors targeting one form of this enzyme are very likely to also prove effective against the other. The key features of these sites are summarised in the proposed generic *Plasmodium *PNP mechanism shown in Figure 6 and in the schematic in Figure 7.

Although correlation of the active site details of the *Pf*PNP-HRA complex with the expected mechanism is not straightforward, several characteristics suggest the complex is more indicative of a post-transition intermediate conformation along the reaction coordinate rather than a true product complex. In particular, the ribose C1' is located close to equidistant between the base N9 and arsenate O1, unlike in previous complexes of *Pf*PNP. In this respect the *Pf*PNP-HRA complex is likely to form a good representation of the point of atomic symmetry along the reaction coordinate, and as such may provide a valuable addition to structure-based design efforts.

Despite the remarkably close similarity between *Pf*PNP and *Pv*PNP, the response of each enzyme to the 2'-deoxyinosine substrate is differentiated by the observation of an unusual form of substrate activation in *Pv*PNP. Although the mechanism by which this arises is currently unclear, this observation demonstrates that even virtually indistinguishable active sites can respond differently to some substrates – possibly because of electrostatic effects from peripheral regions. Inhibitors that resemble 2'-deoxyinosine might therefore prove less effective against *Pv*PNP. Subtle differences such as this may need to be considered during enzyme inhibitor development.

## Methods

### Preparation of recombinant *Plasmodium *PNPs – cloning, expression, purification

The gene for *Pf*PNP was exponentially amplified by PCR from genomic DNA and inserted into the pET28a expression vector (Novagen) using procedures essentially as previously described by [9]. Amplification of the *Pv*PNP gene was performed using the same method with the specific primers: – sense: 5'-TCATCCATGGAAGGCGAAATGCAGAGGC-3', and antisense: 5'-CACACTCGAGGTACTTCTTCGCCAATCGGGC-3'. The resulting gene fragment was inserted into the same expression plasmid using the *Nco*I and *Xho*I restriction sites. Both plasmids were transformed into *E. coli *strain BL21 (DE3) cells (Novagen) for expression. Over-expressed proteins were isolated by nickel affinity chromatography followed by gel filtration using Superdex™ 75 in 150 mM NaCl, 50 mM HEPES, pH 7.5. Protein fractions (>95% purity determined by SDS-PAGE analysis) were concentrated to 10 mg/ml using Vivaspin concentrators (Vivascience) in 100 mM NaCl, 100 mM HEPES, pH 7.5.

### Crystallisation and structure determination of *Pv*PNP and the *Pf*PNP-complex

Crystallisation of both PNP enzymes was achieved by vapour diffusion, with conditions established by sparse matrix crystallisation screens, Crystal Screen1™ and Crystal Screen2™ (Hampton Research). Viable crystals of *Pv*PNP could only be obtained in the absence of nucleosides. The optimised conditions for crystal growth were 5 mg/ml (0.18 mM) *Pv*PNP, 18% PEG 4 K, 0.2 M LiSO_4_, and 0.1 M Tris-HCl, pH 8.5. Crystals of the *Pf*PNP-hypoxanthine-ribose-arsenate (*Pf*PNP-HRA) complex were obtained by pre-incubating10 mg/ml (0.37 mM) *Pf*PNP, 5 mM inosine, and 0.1 M arsenic acid, followed by crystallisation using 4.0 M sodium formate (adjusted to neutral pH) as precipitant.

Diffraction data were collected at the Daresbury SRS synchrotron, station MAD10.1, using monochromatic radiation at wavelengths 1.0745 Å and 1.196 Å respectively for *Pv*PNP and the *Pf*PNP complex. Data were processed using the HKL2000 suite [37] and are summarized in Table 1. Both structures were solved by molecular replacement using the Phaser program [38] in the CCP4 suite [39]. Search models were (1) a monomer of the *Pk*PNP crystal structure (PDB: 2B94) and (2) a monomer (chain A) of the *Pf*PNP-ImmH complex crystal structure (PDB: 1NW4, [6]), in each case with ligands and solvent molecules removed. The resulting structure solutions were refined using REFMAC5 [39] with manual rebuilding in COOT [40]. For the *Pf*PNP complex, TLS refinement was introduced at the very last refinement step using a tls tensor file calculated from the program TLSMD [41]. Completed structures were verified for geometric correctness with MolProbity [42] and SFCHECK [43]. Refinement statistics are also summarized in Table 1.

Coordinates and structure factors have been deposited in the Protein Data Bank (accession codes: *Pv*PNP-SO_4_: [PDB:3EMV]; *Pf*PNP-HRA complex: [PDB:3ENZ]).

### Enzymatic properties of *Pv*PNP compared with *Pf*PNP

Kinetic assays of the *Plasmodium *PNPs with various substrates were based on the forward reaction, in which phosphorolysis of the substrates was catalysed by the enzyme in the presence of phosphate, using a coupled reaction with xanthine oxidase and conditions as described previously [9]. The concentration of desalted xanthine oxidase (Sigma) for the coupled reaction in the inosine and 2'-deoxyinosine assays was 90 mill-units/ml, and substrates were included in the following range of concentrations: inosine (Calbiochem) 0.75 – 3000 μM, 2'-deoxyinosine (Biochemika) 0.75 – 3000 μM, guanosine (Sigma) 0.39–150 μM, 2'-deoxyguanosine (Sigma) 1.71–200 μM and 2-amino-6-mercapto-7-methylpurine riboside (MESG, a component of EnzCheck Phophate Assay Kit, Molecular Probes, Invitrogen) 0.5–500 μM. Measured initial rates were fitted to the classic Michaelis-Menten equation and used for calculation of *K*_M_, *k*_*cat *_and *k*_*cat*_/*K*_M' _with appropriate corrections made for background rates, using the nonlinear regression facility in the GraphPad Prism software (GraphPad Software, Inc., San Diego, USA). For analysis of the substrate activated form of PvPNP with 2'-deoxyinosine (see discussion), the binding sequence could be described as:



where E = enzyme, S = substrate and the rate of the reaction is:

(1)

and the Michaelis constants, *K*_M_, can be approximated as:



To simplify the equation for the analysis software, equation 1 was divided by [E]/[E]:



And substituting the expressions for the Michaelis constants gives:



This was then multiplied by *K*_M1_/*K*_M1 _and by E_0_:



as *V*_max _= *k*·E_0_, equation 2 can be derived and to which the data were fitted:

(2)

## Authors' contributions

AC performed all of the experimental studies and helped to draft the manuscript. RLB participated in the design of the study, the structural interpretations and comparisons, and prepared the manuscript. Both authors read and approved the final manuscript.

## Supplementary Material

Additional file 1 **Steady-state kinetic data for five purine nucleoside substrates for recombinant PNP enzymes**. Table lists enzymatic constants derived in the current study, together with previously published data. Values for *k*_cat _assume one catalytic site per subunit; values for K_M1_, K_M2_, k_cat1 _and k_cat2 _for 2'-Deoxyinosine refer to Equation 2 in Methods.; Other values from ^*a *^[8], ^*b *^[21], ^*c *^[22], ^*d *^[23], ^*e *^[24], and^*f *^[25]. Click here for file
