# Supplementary material for: Conservation of structure and activity in Plasmodium purine nucleoside phosphorylases
Source: BMC Struct Biol. 2009 Jul 3;9:42. doi: 10.1186/1472-6807-9-42 (PMC2721837; doi:10.1186/1472-6807-9-42)
Supplement: Additional file 1 — Steady-state kinetic data for five purine nucleoside substrates for recombinant PNP enzymes. Table lists enzymatic constants derived in the current study, together with previously published data. Values for kcat assume one catalytic site per subunit; values for KM1, KM2, kcat1 and kcat2 for 2'-Deoxyinosine refer to Equation 2 in Methods.; Other values from a [8], b [21], c [22], d [23], e [24], andf [25]. [file 1472-6807-9-42-S1.doc]

**Additional file 1 - Steady-state kinetic data for five purine nucleoside substrates for recombinant PNP enzymes**.

Table lists enzymatic constants derived in the current study, together with previously published data.

| **Substrates** | **Parameters** | ***Pv*PNP** | ***Pf*PNP** | **Human PNP** | ***Tg*PNP** *^e^* | ***Ag*PNP** *^f^* | ***Ec*PNP** *^a^* |
| --- | --- | --- | --- | --- | --- | --- | --- |
| Inosine | *K*_M_ (µM) | 15.4 ± 0.7 | 4.7 ± 0.2 (4.7 *^c^*) | 40 *^a^* | 13.1 | 585 | 70 |
|  | *k*_cat_ (s^-1^) | 1.2 ± 0.01 | 1.1 ± 0.01 (1.1 *^c^*) | 56 *^a^* | 2.6 | 41 | 88 |
|  | *k*_cat_/*K*_M_ (s^-1^M^-1^) | 7.8 x 10^4^ ± 1.3 x 10^4^ | 2.4 x 10^5^ ± 0.4 x 10^5^  (2.3 x 10^5^ *^c^*) | 1.4 x 10^6^ *^a^* | 1.98 x 10^5^ | 7.0 x 10^4^ | 1.3 x 10^6^ |
| Guanosine | *K*_M_ (µM) | 11.2 ± 0.5 | 2.2 ± 0.1 (9.4 *^c^*) | 12 *^a^* | 9.4 | 187 | 20 |
|  | *k*_cat_ (s^-1^) | 0.7 ± 0.01 | 1.1 ± 0.01 (2.6 *^c^*) | 26 *^a^* | 3.6 | 1.0 | 59 |
|  | *k*_cat_/*K*_M_ (s^-1^M^-1^) | 6.1 x 10^4^ ± 1.9 x 10^4^ | 4.9 x 10^5^ ± 1.3 x 10^5^  (2.8 x 10^5^ *^c^*) | 2.3 x 10^6^ *^a^* | 3.83 x 10^5^ | 5.0 x 10^3^ | 3.0 x 10^6^ |
| 2**′**-Deoxyinosine | *K*_M_ (µM) | 61.4 ± 13.7  28.5 ± 8.5* | 25.3 ± 0.9 (91 *^c^*) | 45 *^b^* | 259 | 190 | 180 |
|  | *k*_cat_ (s^-1^) | 5.6 ± 0.95  1.2 ± 0.01* | 1.5 ± 0.01 (0.9 *^c^*) |  | 0.48 | 54 | 231 |
|  | *k*_cat_/*K*_M_ (s^-1^M^-1^) | 9.1 x 10^4^ ± 6.9 x 10^4^  4.4 x 10^4^ ± 0.2 x 10^4^ * | 5.9 x 10^4^ ± 1.0 x 10^4^  (9.8 x 10^3^ *^c^*) |  | 1.85 x 10^3^ | 2.8 x 10^5^ | 1.3 x 10^6^ |
| 2**′**-Deoxyguanosine | *K*_M_ (µM) | 35.1 ± 1.8 | 16.3 ± 0.7 (69 *^a^*) | 38 *^c^* | - | 50 | - |
|  | *k*_cat_ (s^-1^) | 0.7 ± 0.01 | 1.1 ± 0.02 (0.2 *^a^*) | 48 *^c^* | - | 5.4 | - |
|  | *k*_cat_/*K*_M_ (s^-1^M^-1^) | 2.1 x 10^4^ ± 0.7 x 10^4^ | 6.9 x 10^4^ ± 2.0 x 10^4^  (3.0 x 10^3^ *^a^*) | 1.3 x 10^6^ *^c^* | - | 1.1 x 10^5^ | - |
| MESG | *K*_M_ (µM) | 42.3 ± 3.4 | 24.5 ± 1.9 | 358 *^d^* | - | - | - |
|  | *k*_cat_ (s^-1^) | 3.6 ± 0.09 | 4.2 ± 0.08 | 40 *^d^* | - | - | - |
|  | *k*_cat_/*K*_M_ (s^-1^M^-1^) | 8.5 x 10^4^ ± 2.5 x 10^4^ | 1.7 x 10^5^ ± 0.4 x 10^5^ | 1.1 x 10^5^ *^d^* | - | - | - |

Footnote: Values for *k*_cat_ assume one catalytic site per subunit; values for K_M1_,K_M2_, k_cat1_ and k_cat2_ for 2**′**-Deoxyinosine refer to Equation 2 in Methods.; Other values from *^a^*  [7], *^b^*  [40], *^c^* [20], *^d^*  [41], *^e^*  [42], and *^f^*  [43].
